# Supplementary material for: Measuring single cell divisions in human tissues from multi-region sequencing data
Source: Nat Commun. 2020 Feb 25;11:1035. doi: 10.1038/s41467-020-14844-6 (PMC7042311; doi:10.1038/s41467-020-14844-6)
Supplement: Supplementary file 2 — Supplementary Information [file 41467_2020_14844_MOESM2_ESM.pdf]

Supplementary Information for:

Measuring single cell divisions in human tissues from multi-region sequencing data

Benjamin Werner[1,2,*], Jack Case[1,3], Marc J. Williams[4,5,6], Kate Chkhaidze[1], Daniel Temko[4], Javier Fernandez-Mateos[1], George D. Cresswell[1], Daniel Nichol[1], William Cross[4], Inmaculada Spiteri[1], Weini Huang[7,8], Ian Tomlinson[9], Chris P. Barnes[5,10], Trevor A. Graham[4,*] & Andrea Sottoriva[1,*]

[1]Evolutionary Genomics & Modelling Lab, Centre for Evolution and Cancer, The Institute of Cancer Research, Sutton, London SM2 5NG, UK

[2]Evolutionary Dynamics Group, Centre for Cancer Genomics & Computational Biology, Barts Cancer Institute, Queen Mary University of London, Charterhouse Square, London, UK EC1M 6BQ.

[3]University of Cambridge

[4]Evolution and Cancer Laboratory, Centre for Cancer Genomics & Computational Biology, Barts Cancer Institute, Queen Mary University London, London, Charterhouse Square, London, UK EC1M 6BQ.

[5]Department of Cell and Developmental Biology, University College London, London, UK

[6]Centre for Mathematics and Physics in the Life Sciences and Experimental Biology (CoMPLEX), University College London, London, UK

[7]Group of Theoretical Biology, The State Key Laboratory of Biocontrol, School of Life Science,

Sun Yat-sen University, Guangzhou, 510060 China

[8]School of Mathematical Sciences, Queen Mary University London, London, UK.

[9]Institute of Cancer and Genomic Sciences, University of Birmingham, Birmingham, UK

[10]UCL Genetics Institute, University College London, London, UK

*Correspondence should be addressed to: b.werner@qmul.ac.uk, t.graham@qmul.ac.uk, andrea.sottoriva@icr.ac.uk

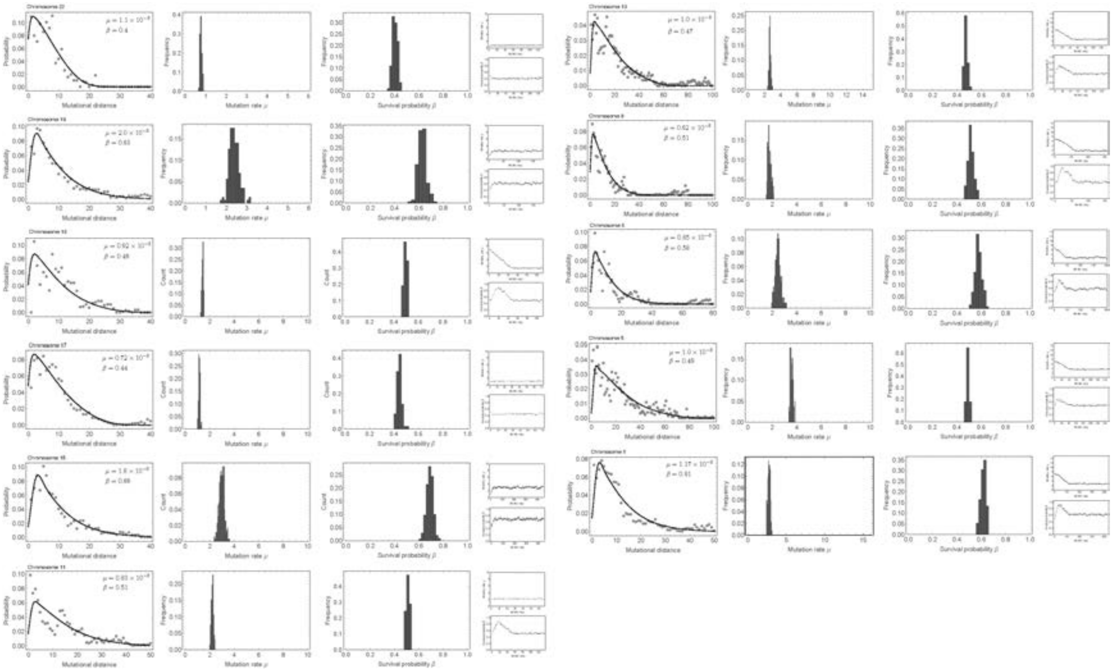

Supplementary Figure 1: Patient 02 mutational distance distribution and MCMC parameter inference per chromosome. Chromosomes with sub-clonal copy number alterations were discarded from the analysis. Original data taken from[1].

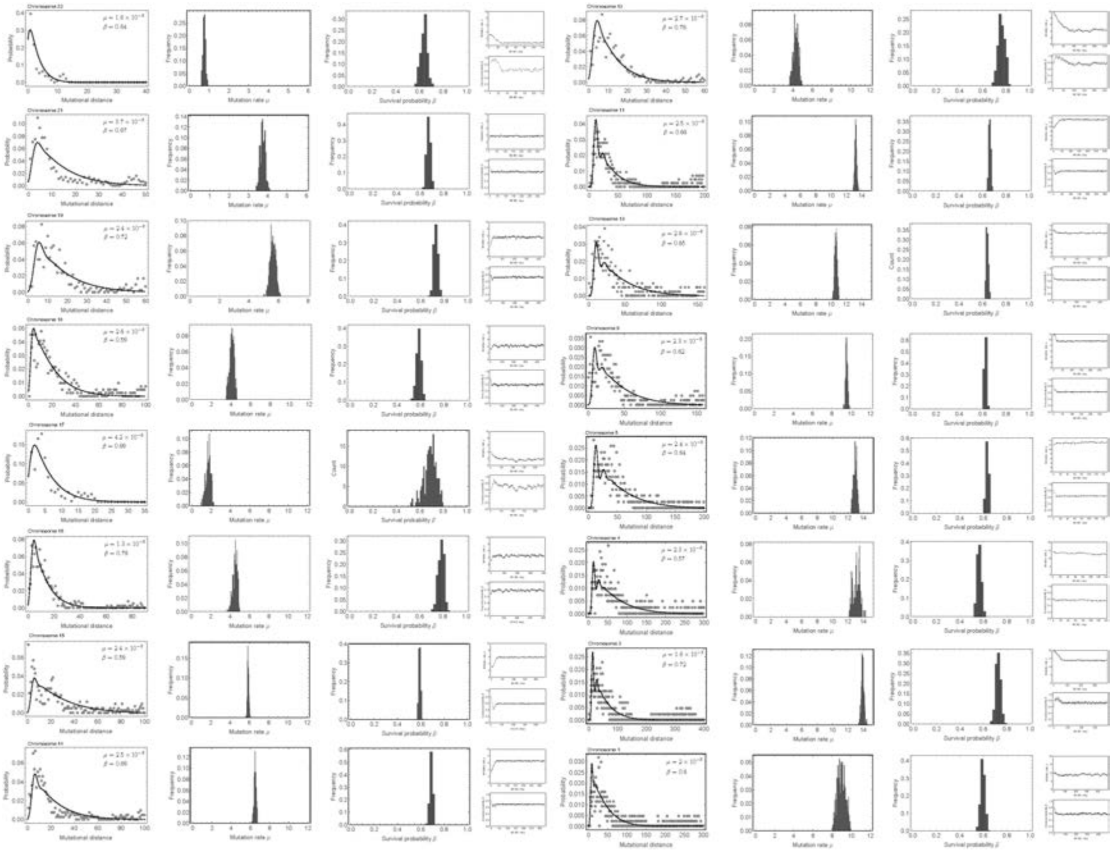

Supplementary Figure 2: Patient 03 mutational distance distribution and MCMC parameter inference per chromosome. Chromosomes with sub-clonal copy number alterations were discarded from the analysis. Original data taken from[1].

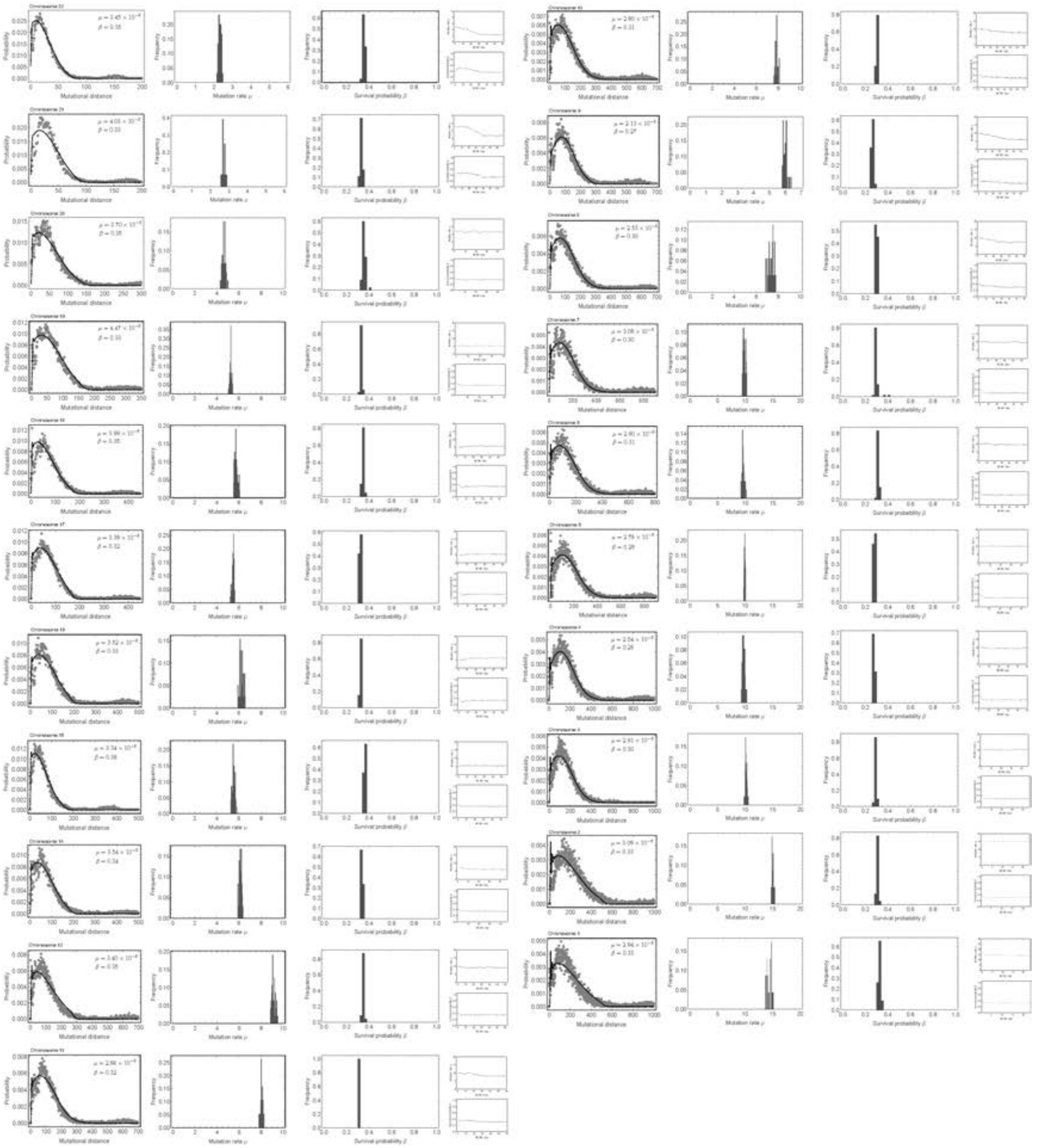

Supplementary Figure 3: Patient 04 mutational distance distribution and MCMC parameter inference per chromosome. Chromosomes with sub-clonal copy number alterations were discarded from the analysis. Original data taken from[1].

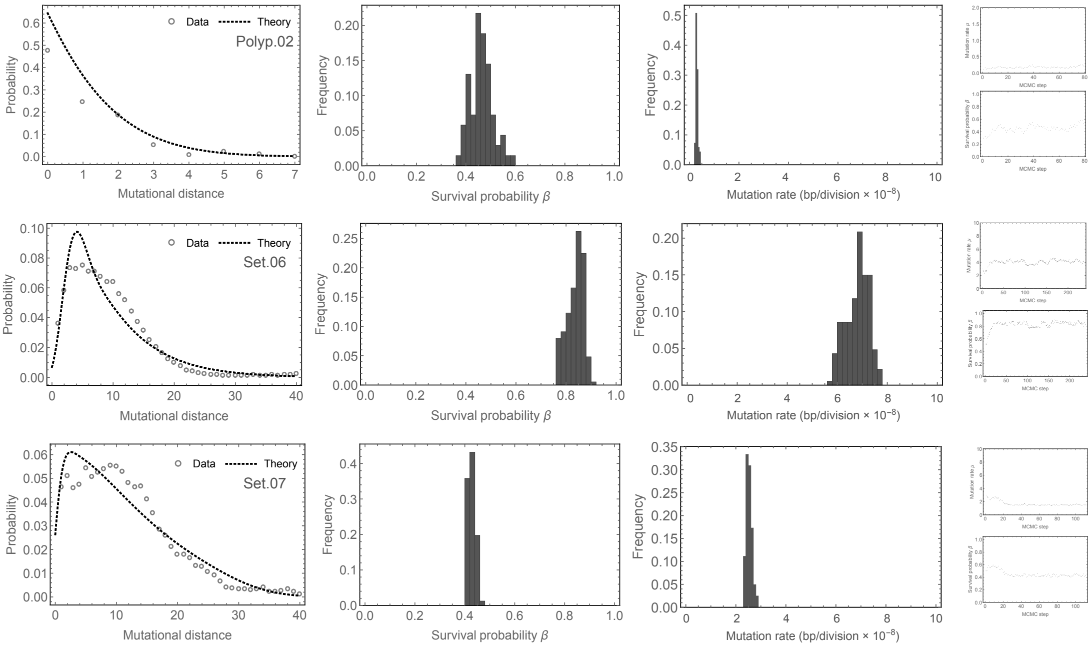

Supplementary Figure 4: Mutational distance distribution and MCMC inference from a single exome sequenced adenoma (top) and two exome sequenced carcinomas (bottom). Note, the adenoma shows smaller mutational differences between ancestral cells compared to both carcinomas and presents with a near normal mutation rate. However the per-cell survival probability is higher compared to normal tissue and the adenoma is expected to clonally expand.

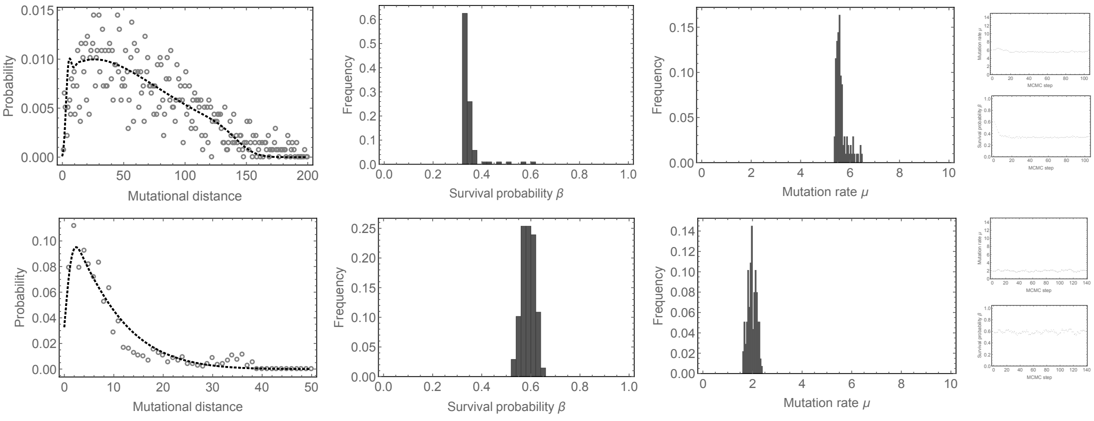

Supplementary Figure 5: Mutational distance distribution and MCMC inference from 2 TracerX patients[2]. Note, the first case has an approximately 5 times increased distance of mutational distances compared to most colon, renal and lung cases analysed here. Together with the MSI colorectal cancer this patient has the highest mutation rate per cell division.

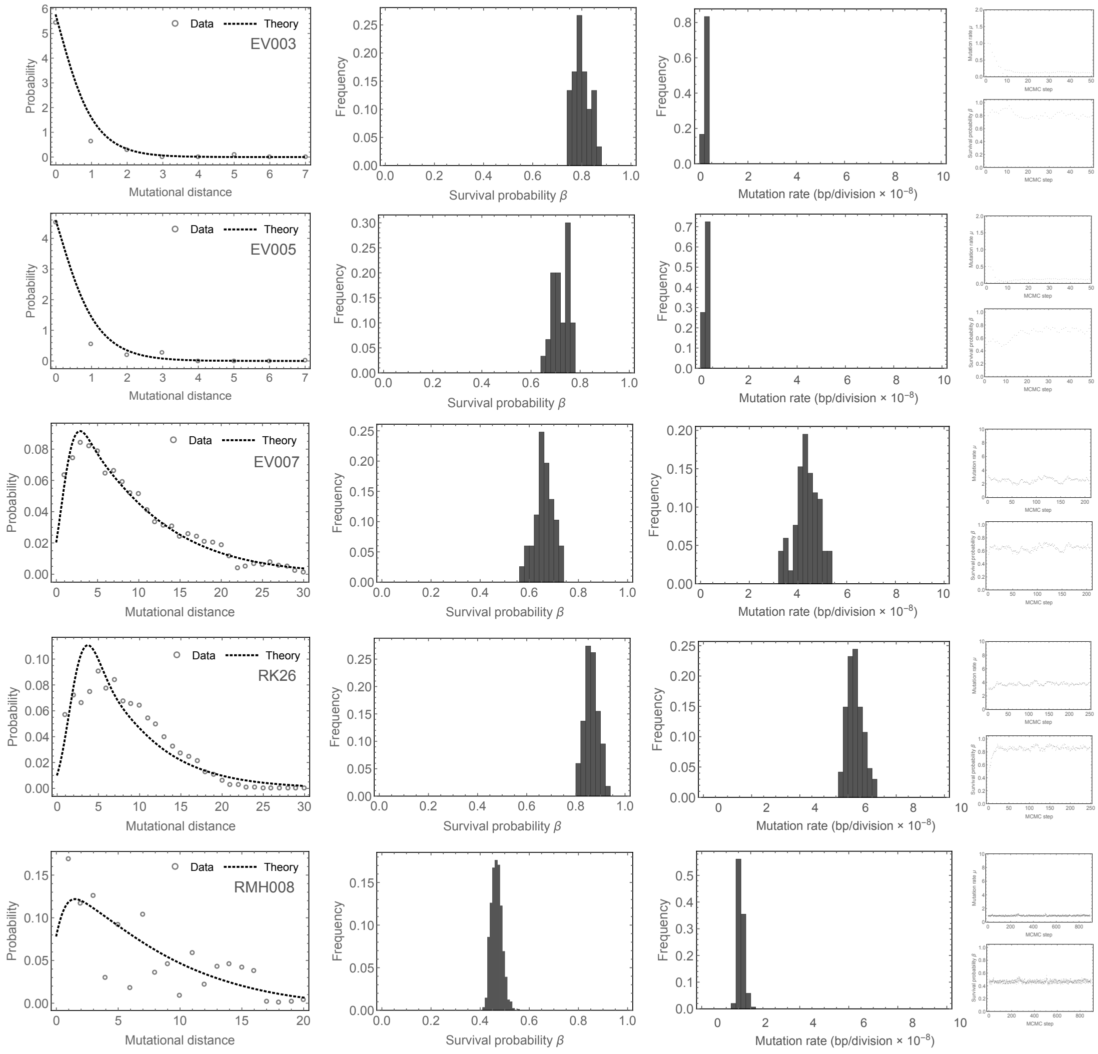

Supplementary Figure 6: Mutational distance distribution and MCMC inference from 5 exome sequenced renal cell carcinomas[3]**.** Surprisingly, two renal cell carcinomas appear to have near normal mutation rates (EV003 and EV005), similar to the colon adenoma. However, all 5 cases present with high per cell survival probabilities.

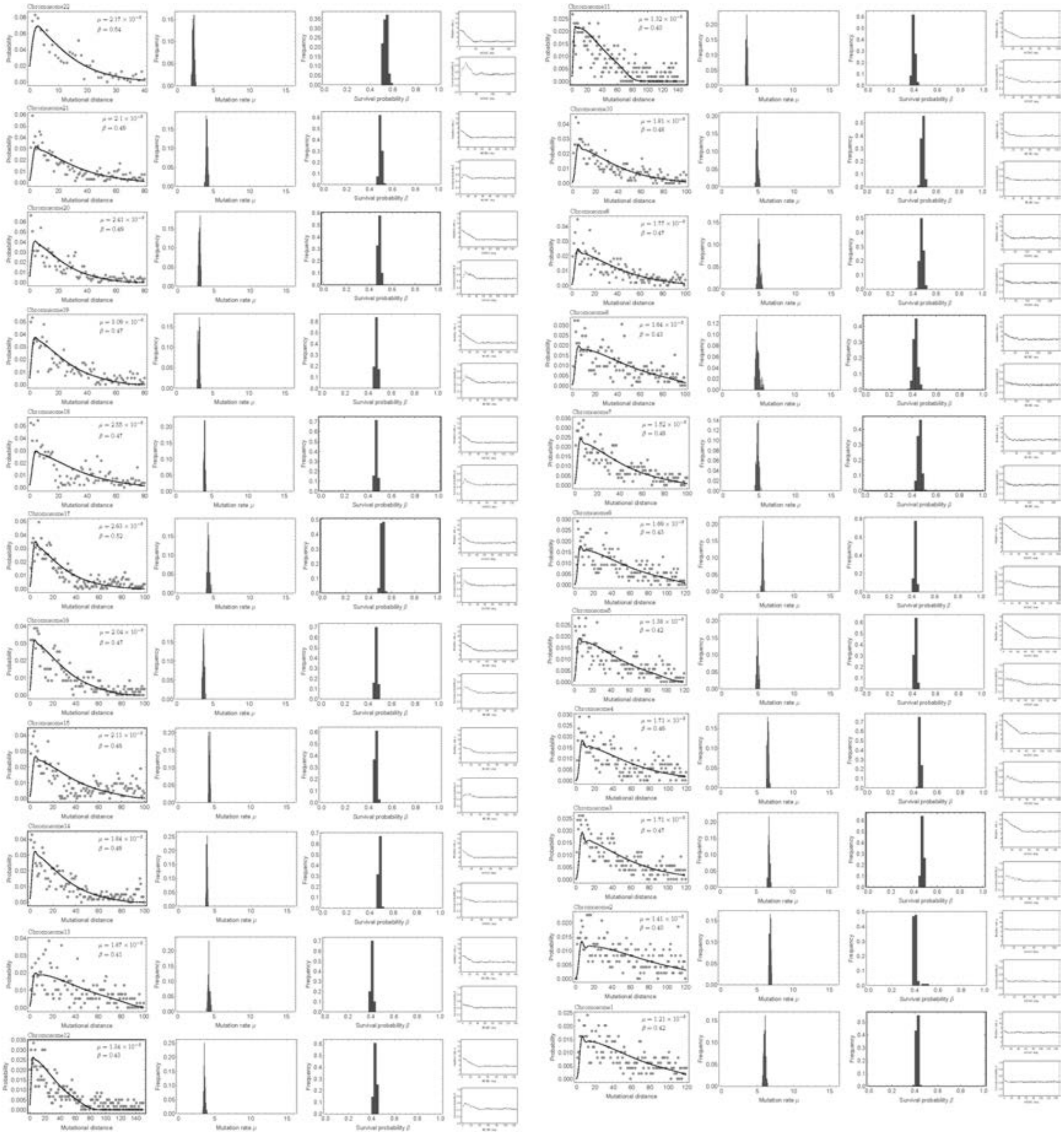

Supplementary Figure 7: Mutational distance distribution and MCMC inference for individual chromosomes inferred from 7 whole genome sequenced samples of a MSI+ colon cancer patient. Data was taken from[4].

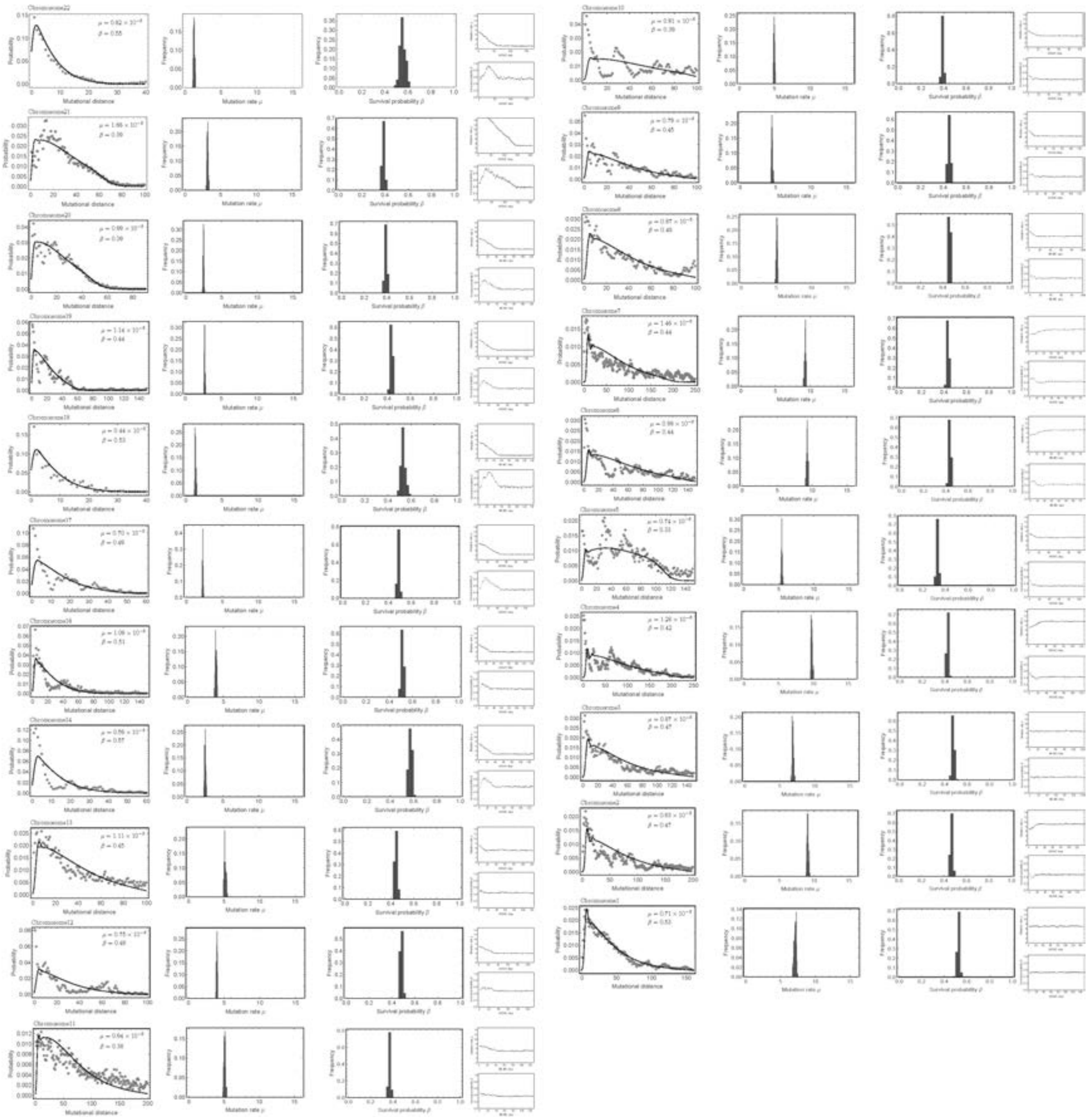

Supplementary Figure 8: Mutational distance distribution and MCMC inference for individual chromosomes inferred from 9 whole genome sequenced samples of a MSS colon cancer patient. Data was taken from[4].

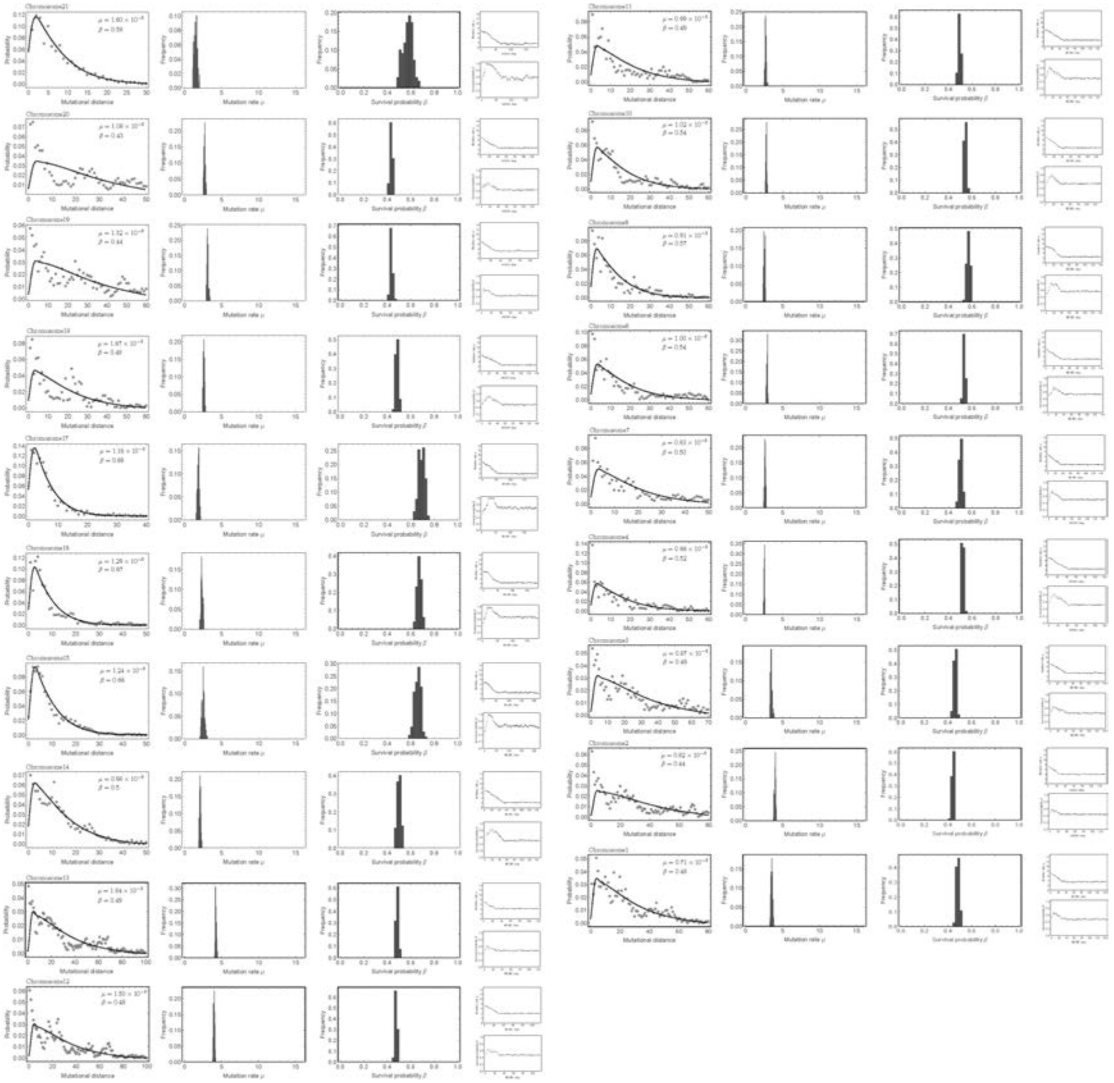

Supplementary Figure 9: Mutational distance distribution and MCMC inference for individual chromosomes inferred from 9 whole genome sequenced samples of a MSS colon cancer patient. Data was taken from[4].

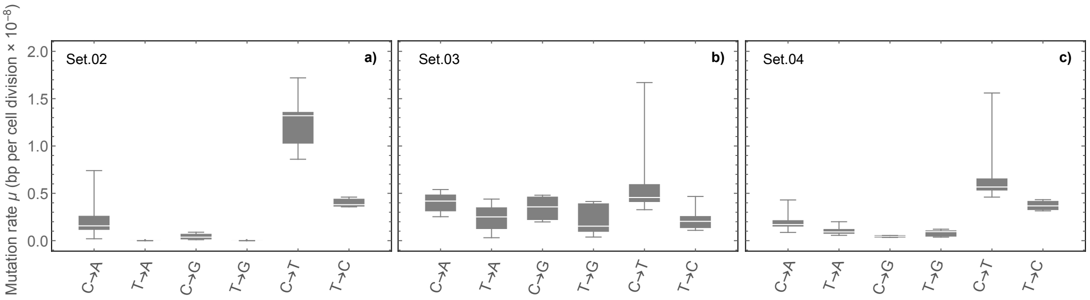

Supplementary Figure 10: Mutation rates of mutational subtypes. a)-c) The mutation rate for mutational subtypes was inferred based on our MCMC algorithm for individual chromosomes (see Figure 5 in the main text) for all 3 patients separately and normalised for the C & T content at each chromosome. Inferences for Patient 02 are based on independent measures of $n = 11$ chromosomes, in Patient 03 on independent measures of $n = 16$ chromosomes and in Patient 04 on independent measures of $n = 21$ chromosomes. The box plots are defined as centre = mean, bounds of box = 25% and 75% quantile and maxima = $Q_3 + 1.5 \times \text{IQR}$ and minima = $Q_1 - 1.5 \times \text{IQR}$. In Patient 02 and 04 transitions show higher mutation rates than transversions. Interestingly, in Patient 02 transversions T → A and T → G are absent, whereas in Patient 04 they are detectable. Patient 03 shows a distinct pattern of mutation accumulation. Here transitions and transversions appear equally likely, with C → X mutations slightly more likely compared to T → X mutations.

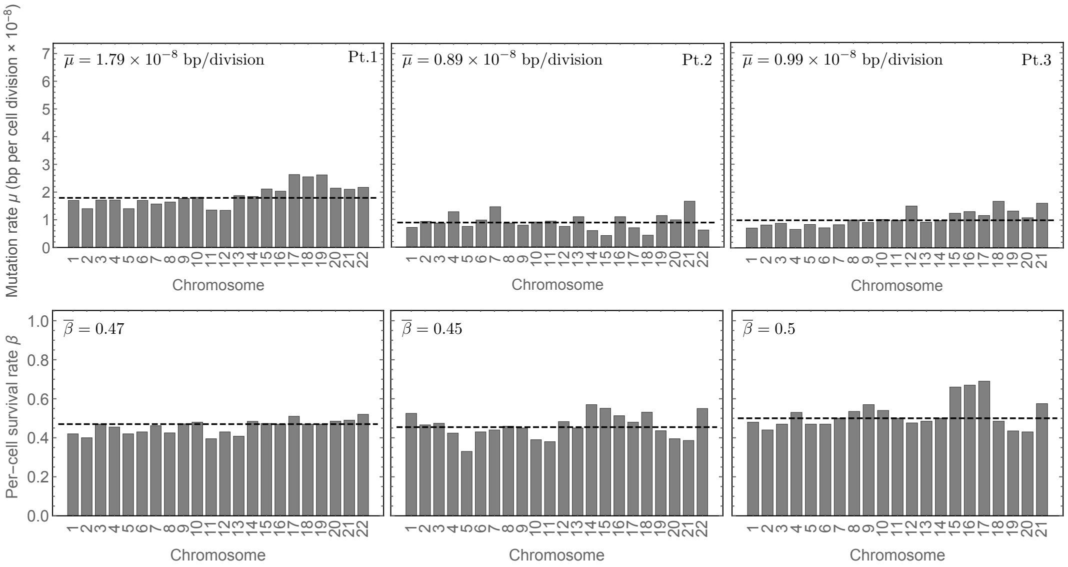

Supplementary Figure 11: **Inference of the mutation rate μ per cell division and the per-cell survival rate β per chromosome for the three patients shown in SI Figures 9-11.** Insets show median mutation and per-cell survival rates. Data was taken from[4].

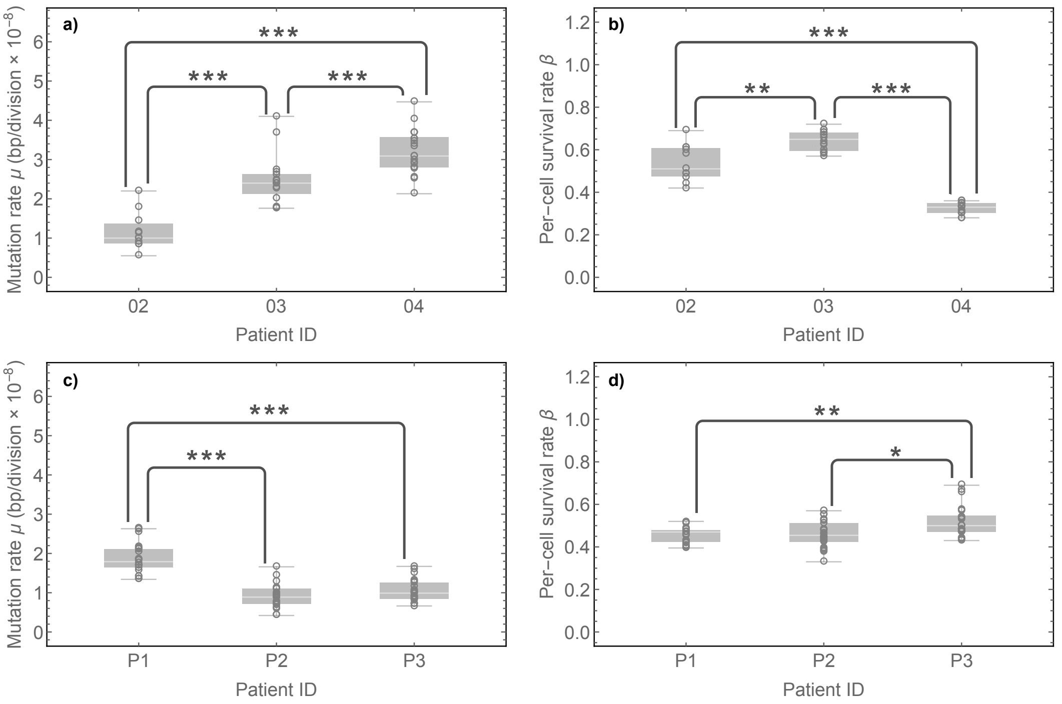

Supplementary Figure 12: Between patient differences of evolutionary parameters. Shown are the inferences of the mutation and per-cell survival rates per chromosome (dots) for 6 whole genome sequenced colorectal cancers. Panels **(a)** and **(b)** show the cohort of Cross et al. and panels **(c)** and **(d)** the original patients by Roerink et al. Inferences for Patient 02 are based on independent measures of $n = 11$ chromosomes, in Patient 03 on independent measures of $n = 16$ chromosomes and in Patient 04 on independent measures of $n = 21$ chromosomes. Inferences for Patient P1 $n = 22$ for Patient P2 $n = 21$ and for Patient P3 $n = 19$ independent measures of chromosomes. Box plots are defined as centre = mean, bounds of box = 25% and 75% quantile and maxima $= Q_3 + 1.5 \times \mathrm{IQR}$ and minima $= Q_1 - 1.5 \times \mathrm{IQR}$. A two-sided Mann-Whitney-U-test was used to test between patient differences, symbols correspond to the short notation: $* = p < 0.01$, $** = p < 0.001$ and $*** = p < 0.0001$.

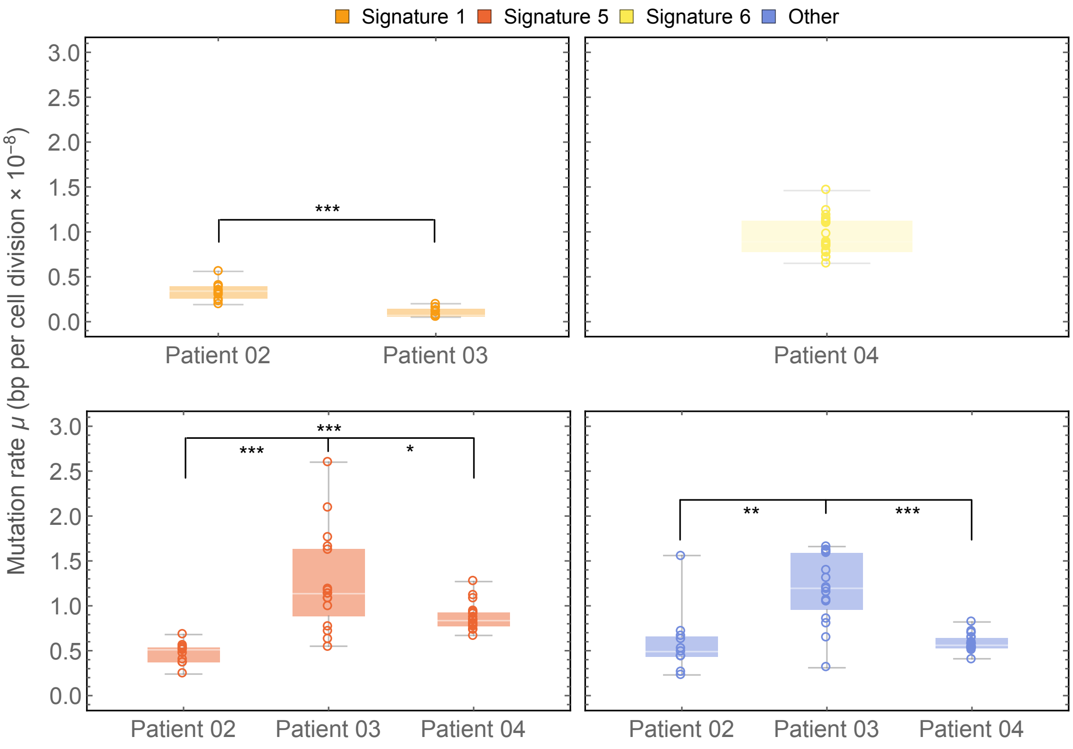

Supplementary Figure 13: Distribution of mutational signature mutation rate per chromosome for Patients 02-04. Mutation rates per cell division of mutational signatures differ significantly between patients. Inferences for Patient 02 are based on independent measures of $n = 11$ chromosomes, in Patient 03 on independent measures of $n = 16$ chromosomes and in Patient 04 on independent measures of $n = 21$ chromosomes. Box plots are defined as centre = mean, bounds of box = 25% and 75% quantile and maxima = $Q_3 + 1.5 \times$ IQR and minima = $Q_1 - 1.5 \times$ IQR. (*: p<0.05, ** :p<0.01, ***: p<0.001, two-sided Mann-Whitney-U-test).

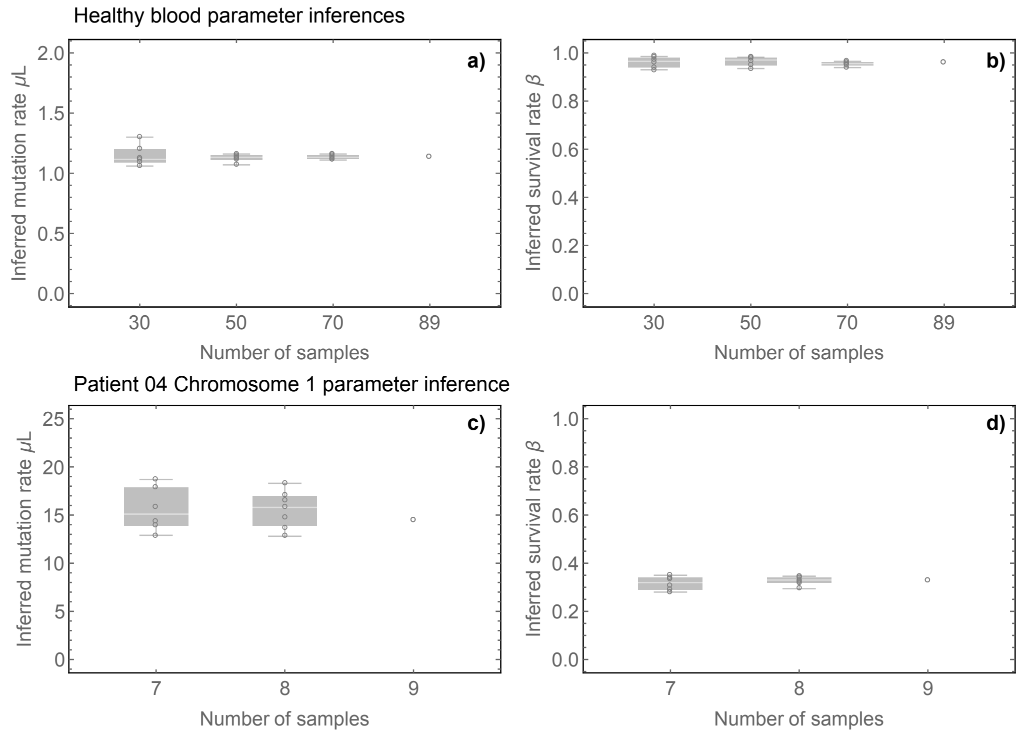

Supplementary Figure 14: Data down-sampling and parameter inferences. a),b) Parameter inferences for the down-sampled data of healthy haematopoiesis. **c),d)** Parameter inferences for the down-sampled data of chromosome 1 of patient 04. Each Boxplot shows $n = 10$ independent MCMC inferences of randomly down-sampled data. Box plots are defined as centre = mean, bounds of box = 25% and 75% quantile and maxima = $Q_3 + 1.5 \times \text{IQR}$ and minima = $Q_1 - 1.5 \times \text{IQR}$

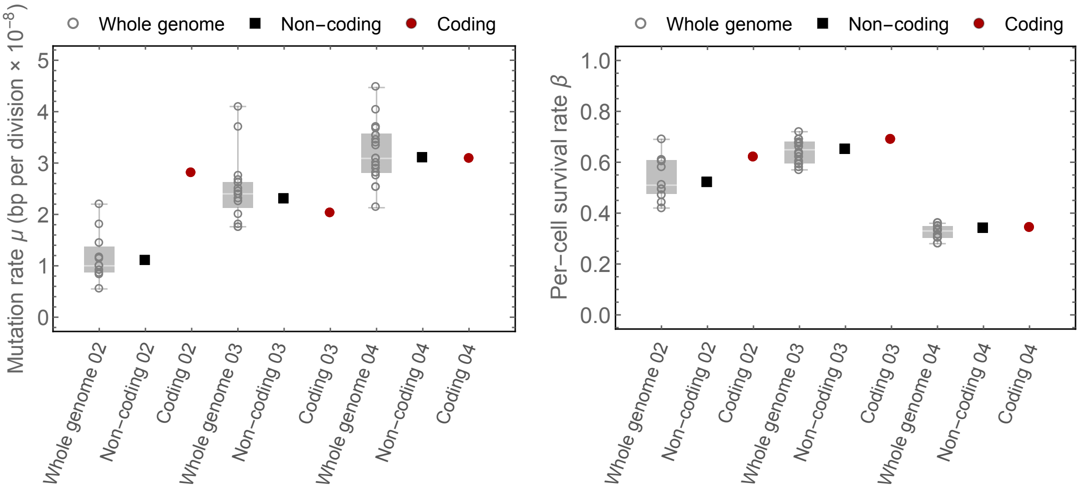

Supplementary Figure 15: Inference of per-cell mutation and per-cell survival rate for whole genome (per chromosome, open grey circles), non-coding (black squares) and coding mutations (red circles) in Patients 02-04. The coding mutation rate in patient 02 is slightly increased compared to whole genome inferences ($\mu_{WG}^{02} = 1 \times 10^{-8}, \mu_{Ex}^{02} = 2.8 \times 10^{-8}$), they are slightly lower in patient 03 ($\mu_{WG}^{03} = 2.4 \times 10^{-8}, \mu_{Ex}^{03} = 2.02 \times 10^{-8}$) and the same in patient 04 ($\mu_{WG}^{04} = 3.1 \times 10^{-8}, \mu_{Ex}^{04} = 3.08 \times 10^{-8}$). Non-coding mutation rates agree with median whole genome mutation rates. Inferences for Patient 02 are based on independent measures of $n = 11$ chromosomes, in Patient 03 on independent measures of $n = 16$ chromosomes and in Patient 04 on independent measures of $n = 21$ chromosomes. Box plots are defined as centre = mean, bounds of box = 25% and 75% quantile and maxima = $Q_3 + 1.5 \times \text{IQR}$ and minima = $Q_1 - 1.5 \times \text{IQR}$.

(*: p<0.05, ** :p<0.01, ***: p<0.001, Mann-Whitney-U-test).

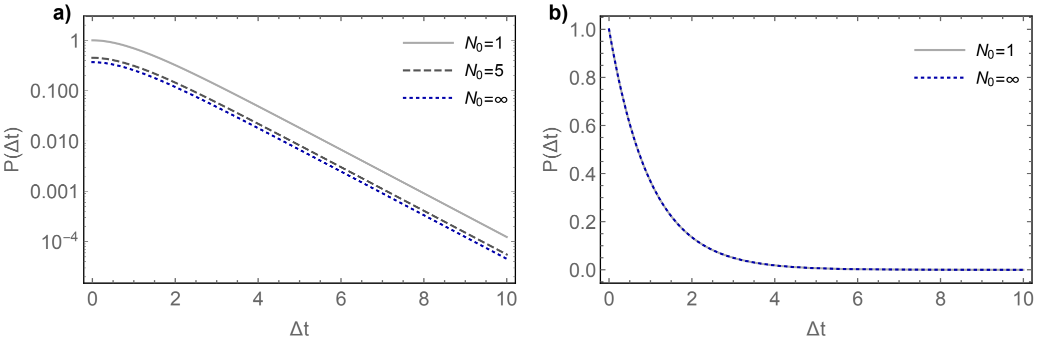

Supplementary Figure 16. Analytical approximation dependence on N_0. a) Realisations of equation 9 for different values of N_0. Here $N_0 = \infty$ corresponds to the approximate expression S3. Note equations 9 and 10 are not normalised. **b)** The normalised equations 10 and 9 are identical for all $N_0 \geq 1$.

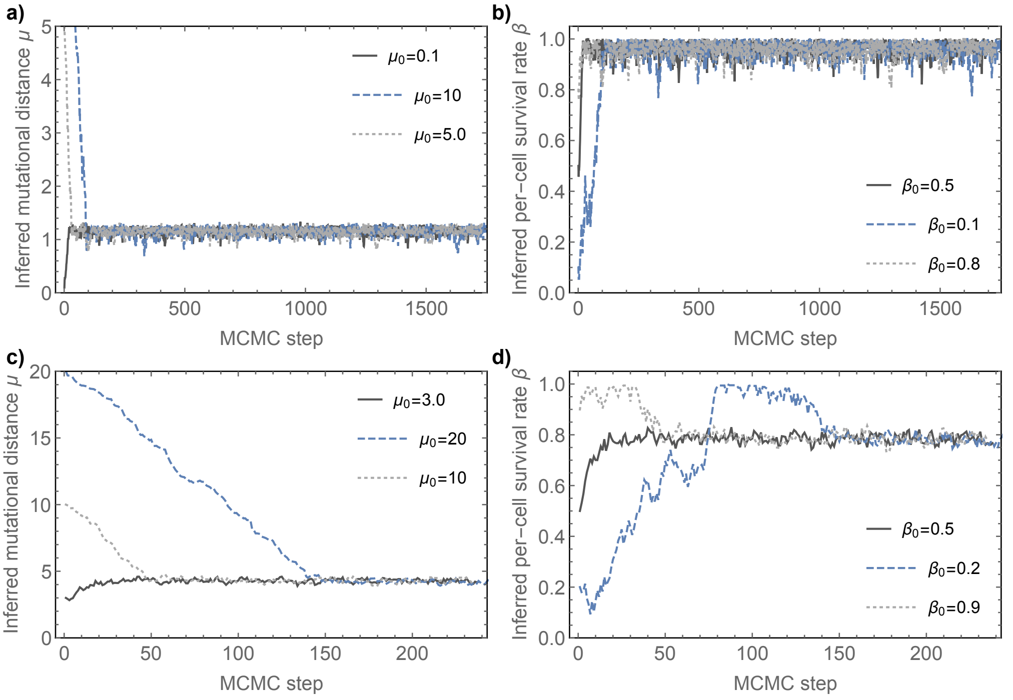

Supplementary Figure 17: Examples of the MCMC parameter estimation. Shown are multiple realisations of the MCMC algorithm for **a) & b)** healthy haematopoiesis (see also Figure 3 in the main text) and **c) & d)** Chromosome 19 of Patient 02 as shown in panel a) of Figure 5 in the main text. Each inference started with different initial conditions μ_0 and β_0. We use a realisation of the Metropolis-Hastings algorithm and chains are converging to stable parameter pairs.

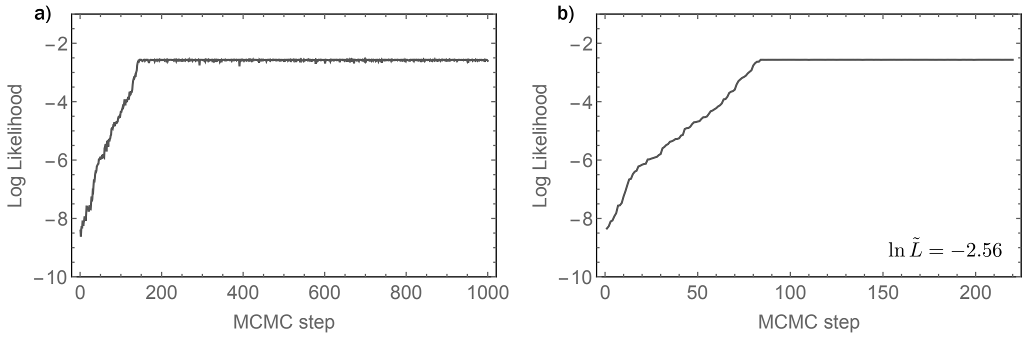

Supplementary Figure 18.:Example of Log Likelihood trace of the MCMC parameter estimation. Shown is the trace of the Log Likelihood function for a) the first 1000 steps of the MCMC algorithm for the parameter inference of healthy haematopoiesis. b) Of the 1000 MCMC steps (panel a), 220 parameter pairs were accepted during this realisation. After a burn-in phase of 100 steps, we find $\log L = -2.56$.

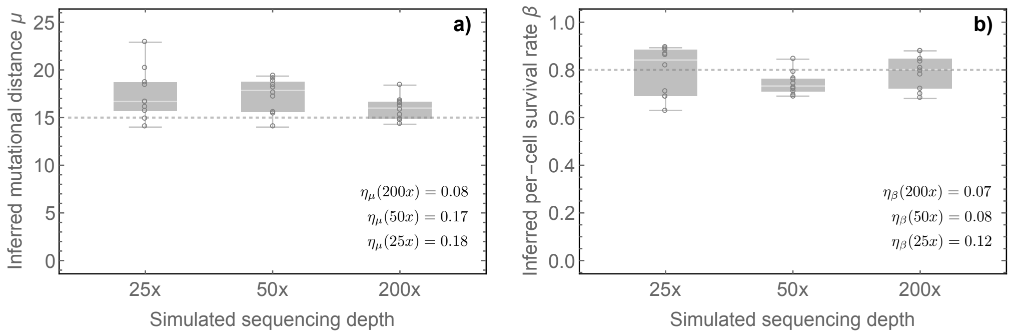

Supplementary Figure 19: Parameter inference for different sequencing depth. Shown are the parameter inferences of the mutation rate **(a)** and the survival rate **(b)** for 10 spatial (2d) tumour simulations with $\mu = 15$ and $\beta = 0.8$ from the mutational distance distribution derived from 9 bulk samples with simulated sequencing depth of 25x, 50x and 200x. Shown are also the relative errors η for each scenario. The construction of the mutational distance distribution relies on the identification of clonal mutations within bulk samples. Consequently, the inferences remain accurate for a simulated sequencing depth of 25x. Boxplots are derived from $n = 10$ independent MCMC inferences on stochastic computer simulations. Box plots are defined as centre = mean, bounds of box = 25% and 75% quantile and maxima = $Q_3 + 1.5 \times \text{IQR}$ and minima = $Q_1 - 1.5 \times \text{IQR}$.

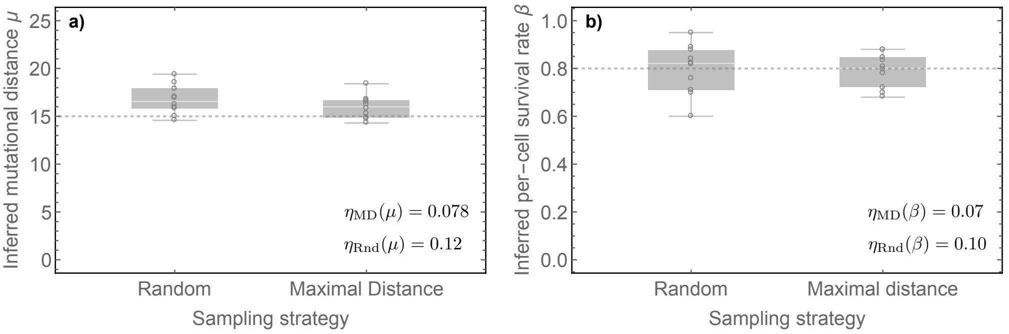

Supplementary Figure 20.: Random and Maximal Distance sampling. In order to test differences of random and maximal distance sampling, we did 10 spatial (2d) simulations with same underlying parameters (dashed lines). We then took 9 bulk samples either randomly or with maximal spatial distance and used our MCMC for parameter inferences. A maximal distance sampling strategy performs slightly better compared to random sampling (indicated by the relative errors η). Sown are **a)** mutation rate and **b)** per-cell survival rate inferences. Boxplots are derived from $n = 10$ independent MCMC inferences on stochastic computer simulations. Box plots are defined as centre = mean, bounds of box = 25% and 75% quantile and maxima = $Q_3 + 1.5 \times \mathrm{IQR}$ and minima = $Q_1 - 1.5 \times \mathrm{IQR}$.

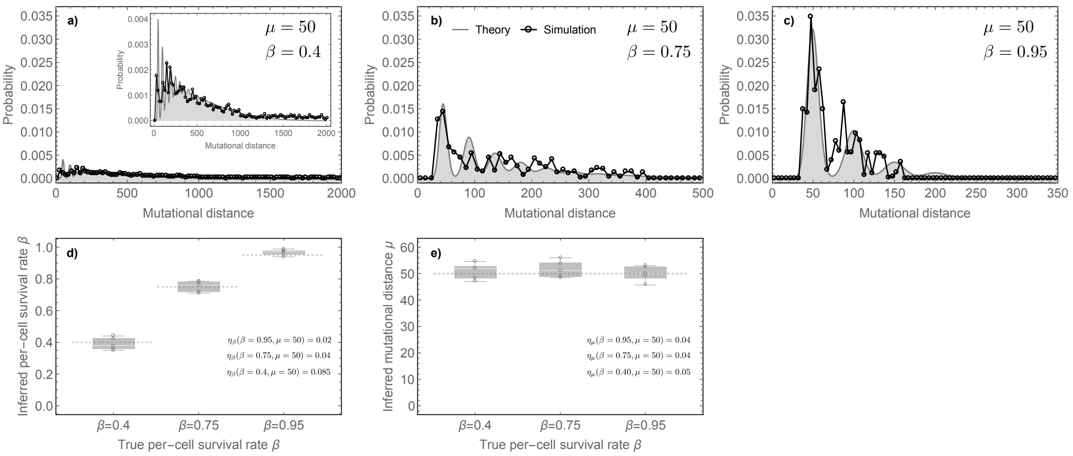

Supplementary Figure 21: Spatial stochastic simulation inferences with varying per-cell survival rates. Panels **(a)-(c)** show examples for the mutational distance distribution reconstructed for cases of high mutation rate and different per-cell survival rates (2d spatial stochastic simulations). The distributions are plotted with same y-axes to show the dramatic differences in the shape of the distributions (notice the different scales of the x-axis in panels a) to c)). The inset of panel **(a)** shows the same distribution, just with a differently scaled y-axis. Panels **(d) & (e)** show the inference of the evolutionary parameters for independent stochastic runs of spatial tumour simulations (9 bulk samples per simulation). Inferences are robust for low and high death as shown by relative errors η. Boxplots are derived from $n = 10$ independent MCMC inferences on stochastic computer simulations. Box plots are defined as centre = mean, bounds of box = 25% and 75% quantile and maxima = $Q_3 + 1.5 \times \mathrm{IQR}$ and minima = $Q_1 - 1.5 \times \mathrm{IQR}$.

References

1. Cross, W. *et al.* The evolutionary landscape of colorectal tumorigenesis. *Nature Ecology & Evolution* 1–14 (2018).
2. Jamal-Hanjani, M. *et al.* Tracking the Evolution of Non–Small-Cell Lung Cancer. *New England Journal of Medicine* **376,** 2109–2121 (2017).
3. Gerlinger, M. *et al.* Genomic architecture and evolution of clear cell renal cell carcinomas defined by multiregion sequencing. *Nature Genetics* **46,** 225–233 (2014).
4. Roerink, S. F. *et al.* Intra-tumour diversification in colorectal cancer at the single-cell level. *Nature* **556,** 457-462 (2018).
